# The Use of Antidepressive Agents and Bone Mineral Density in Women: A Meta-Analysis

**DOI:** 10.3390/ijerph15071373

**Published:** 2018-06-30

**Authors:** Julietta Ursula Schweiger, Ulrich Schweiger, Michael Hüppe, Kai G. Kahl, Wiebke Greggersen, Kamila Jauch-Chara, Eva Fassbinder

**Affiliations:** 1 LADR Zentrallabor Dr. Kramer & Kollegen, Lauenburgerstr. 67, D-21502 Geesthacht, Germany; j.schweiger@ladr.de; 2 Department of Psychiatry and Psychotherapy, Medical School, Lübeck University, Ratzeburger Allee 160, D-23538 Lübeck, Germany; wiebkegreggersen@gmail.com (W.G.); kamila.jauch-chara@uksh.de (K.J.-C.); eva.fassbinder@uksh.de (E.F.); 3 Department of Anesthesiology, Medical School, Lübeck University, Ratzeburger Allee 160, 23538 Lübeck, Germany; michael.hueppe@uni-luebeck.de; 4 Department of Psychiatry, Social Psychiatry and Psychotherapy, Hannover Medical School, Carl-Neuberg-Straße 1, 30625 Hannover, Germany; kahl.kai@mh-hannover.de

**Keywords:** absorptiometry, bone mineral density, antidepressive agents, depressive disorder, meta-analysis, osteoporosis

## Abstract

Antidepressive agents are one of the fastest-growing classes of prescribed drugs. However, the effects of antidepressive agents on bone density are controversial. The aim of this meta-analysis is to evaluate the state of research on the relationship between the use of tricyclic antidepressants (TCAs) or selective serotonin reuptake inhibitors (SSRIs) and bone mineral density (BMD) in women. The database searched was Pubmed. The meta-analysis included human studies in women fulfilling the following criteria: (i) an assessment of bone mineral density in the lumbar spine, the femoral neck or the total hip; (ii) a comparison of the BMD of depressed individuals using antidepressive agents (SSRIs or TCAs), and a control group that did not use antidepressive agents; (iii) measurement of BMD using dual-energy X-ray absorptiometry (DXA); and (iv) calculations of the mean BMD and standard deviation or standard error. Four studies were identified, which, in total, included 934 women using antidepressive agents and 5767 non-using individuals. The results showed that no significant negative composite weighted mean effect sizes were identified for the comparisons between SSRI users and non-users. Similarly, no significant negative composite weighted mean effect sizes were identified for the comparisons between TCA users and non-users, indicating similar BMD in SSRI or TCA users and non-users. The meta-analysis shows that the association between antidepressant medication and bone mineral density has not been extensively researched. Only four studies fulfilled the inclusion criteria. The global result of the literature review and meta-analysis was that the use of antidepressive agents was not associated with lower or higher BMD. This result applies to both SSRIs and TCAs and to all measurement locations (lumbar spine, femoral neck and total hip).

## 1. Introduction

In the United States, antidepressive agents are one of the fastest-growing classes of prescribed drugs [1]. In this context, the rate of treatment during the course of one year has risen from 5.8% in 1996 to 10.1% in 2005 [2]. Approximately 6% of all men and women in the Netherlands used some type of antidepressive agent in 2012 [3]. While the incident prescription of antidepressant drugs has declined slightly since 2000, the overall use of these drugs has increased due to repeated and longer use. The prevalence of the use of antidepressive agents increases with age; it is lowest in 20- to 39-year-olds (3%) and highest in men and women over the age of 80 (10%). The one-year prevalence of antidepressant prescription varies among European countries, ranging from 2.7% in Greece to 15.7% in Portugal [4]. Antidepressive agents are primarily used for the treatment of depressive disorders but are also prescribed for anxiety disorders, sleeping disorders, and pain. However, a considerable proportion of patients (approximately 30%) may receive antidepressant medication without formally fulfilling the criteria for having a mental disorder [5].

Osteoporosis is a highly prevalent degenerative bone disease [6] and is characterized by low bone mineral density and deterioration of the skeletal structure [7]. Osteoporosis interacts with environmental stress factors in increasing the risk of fracture [8]. Osteoporotic fractures increase short-term and long-term mortality [9]. The most common classical risk factors for low bone mineral density (BMD) are low body mass index, a history of fragility fractures, environmental risk, early menopause, smoking, lack of vitamin D, endocrine disorders (insulin-dependent diabetes mellitus, for example), the use of glucocorticoids, excessive alcohol intake, immobility, and depression [10]. Depression is a common chronic mental disorder associated with mood, cognitive, and physical symptoms [11]. Major depressive disorder (MDD) is one of the leading causes of years lived with disability in most countries [12]. The risk of developing MDD is twice as high for women as for men [13]. Several meta-analyses have reported an increased risk of osteoporosis in patients with depression [14,15,16,17,18].

There are many possible mechanisms resulting in low BMD in individuals with depression. Patients with depression show an altered regulation of the hypothalamic-pituitary-adrenal (HPA) system, resulting in hypercortisolism in a substantial proportion of depressed individuals. Underlying this altered regulation is an increased secretion of corticotropin-releasing hormones, corticotropin, and cortisol. The concentrations of glucocorticoid and mineralocorticoid receptors are altered, resulting in decreased feedback effects of cortisol [19]. Cortisol is a well-known factor in bone loss [20]. Proinflammatory cytokines have been related to depressive disorders and studies have shown that depressed patients have high concentrations of proinflammatory cytokines (Interleukin-1, Interleukin-6, tumor necrosis factor alpha and monocyte chemoattractant protein-1) and C-reactive protein (CRP) [21,22]. Cytokines and immune cells may directly stimulate osteoclastic activity [23] and stimulate glucocorticoid secretion via activation of the HPA system. An important feature of MDD is behavioral changes such as high alcohol and nicotine consumption, inadequate nutrition, and low physical activity [24,25,26,27,28,29,30].

The treatment of depression with antidepressive agents such as tricyclic antidepressants (TCAs) or selective serotonin reuptake inhibitors (SSRIs) may also exert negative effects on bone mineral density. In particular, SSRIs have been associated with an increased risk of fractures [31]. The serotonergic pathways in the brain are powerful regulators of bone metabolism [32]. Serotonin receptors (5-HT) and 5-HT transporters (5-HTT) have been identified in many major bone cell types. A polymorphism in the promoter region of the serotonin transporter gene (5-HTTLPR) is assumed to moderate the relationship between stress and depression [33]. There are no data indicating how this polymorphism affects bone mineral density in the general population. SSRIs selectively and potently block the 5-HTT. This results in a higher concentration of free circulating 5-HT, which may have negative effects on bone metabolism. In vitro studies have shown that mice with a null mutation in the 5-HTT gene and mice treated with SSRIs exhibit reduced bone mineral density, altered skeletal architecture, and reduced bone mechanical properties [34,35].

This meta-analysis examines the state of research on the relationship between the use of SSRIs and TCAs and bone mineral density in humans. We are not aware of any other meta-analysis examining this topic. The aim of this cross-sectional study was to perform a meta-analysis of all relevant studies comparing the BMD of patients using SSRIs or TCAs to treat depression to the BMD of an appropriate comparison patient population not using antidepressive agents.

## 2. Methods

### 2.1. Sample of Studies

**Figure 1:**
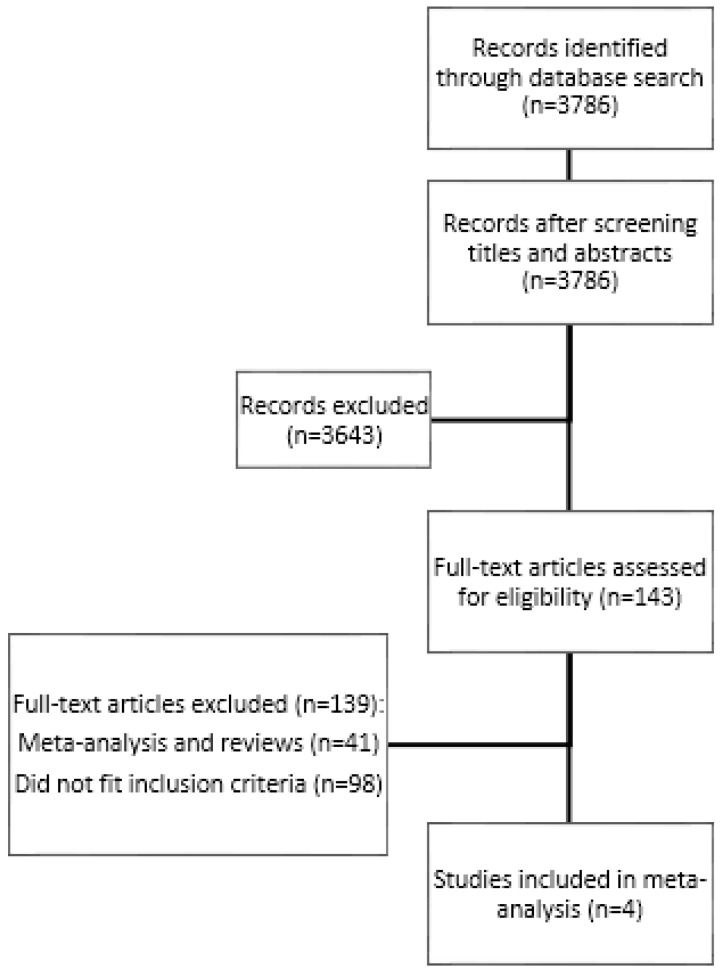
According to the PRISMA statement, the diagram plots the flow of information in the meta-analysis.

This literature review and meta-analysis followed the framework provided by the Preferred Reporting Items for Systematic Reviews and Meta-Analyses (PRISMA) statement [36] (Figure 1). Studies were identified using the Pubmed database, with the search covering the period from the inception of the database to 6 April 2016. A comprehensive literature search using the terms *bone* and *depression* was conducted without language restrictions; results were restricted to human studies. We reviewed each article title and abstract to exclude obviously irrelevant publications. Relevant reports were also double-checked with regard to the reference list of published articles, including several reviews, with no additional records identified. The literature was not restricted to papers with the term “antidepressive agent” because the meta-analysis presented here was part of a bigger project on bone and depression. The inclusion criteria for this specific meta-analysis were (i) an assessment of bone mineral density in the lumbar spine, the femoral neck or the total hip in women; (ii) a comparison of the BMD of depressed women using antidepressive agents (SSRIs or TCAs) and a control group that did not use antidepressive agents; (iii) measurement of BMD using dual-energy X-ray absorptiometry (DXA); and (iv) calculations of the mean BMD and standard deviation or standard error. Studies without differentiation between women and men and only examining fractures without measuring BMD were excluded. (v) It was decided that in the case of a longitudinal study, the data set with the longest exposure to antidepressive agents should be included.

### 2.2. Data Extraction

Data were extracted by two examiners (J.U.S and U.S), using standardized data abstraction forms. The extracted information included (i) the name(s) of the author(s); (ii) the year of publication; (iii) the country in which the study was conducted; (iv) the sample size of the patient and control groups; (v) gender; (vi) age; (vii) menopausal status; (viii) medication; (ix) depression assessment tool; and (x) the BMD, T score and Z score for the lumbar spine, femoral neck and total hip tool.

### 2.3. Statistical Analyses

We conducted several meta-analyses of BMD in users and non-users of antidepressive agents. Analyses were performed with Comprehensive Meta-Analysis (CMA) software (Englewood, NJ, USA). In each meta-analysis, the standardized effect sizes derived from the individual studies were combined to determine a composite mean weighted effect size, along with its 95% confidence interval (CI) and significance level (i.e., the effect size is significant if the CI does not include a zero). Greater weight was given to studies with larger samples; hence, this procedure corrected for the bias of small sample sizes. Because the effects of antidepressant medication on BMD were studied in different settings and because participants’ demographic data differed greatly between studies, we assumed the presence of heterogeneity a priori (i.e., that the effect of individual trials would vary more than expected by chance alone). Therefore, the variance and statistical significance of any differences were assessed with random-effect calculations in all analyses. To determine the validity of the meta-analysis, we employed funnel plots (i.e., plots of the standard difference in means [d] against the standard error of the mean SEM). This step was followed by quantitative evaluation of the degree of asymmetry [37]. The analyses were conducted independently for the following bones: lumbar spine, femoral neck, and total hip. For each bone, all related studies were pooled and individually analyzed for women and men.

## 3. Results

A total of 3786 records were identified through this search. Out of these, 143 full-text articles were assessed for eligibility. In total, four articles complied with the inclusion criteria [38,39,40,41]. These studies included 934 women using antidepressive agents and 5767 non-using individuals (Table 1).

### 3.1. Lumbar Spine

**Figure 2:**
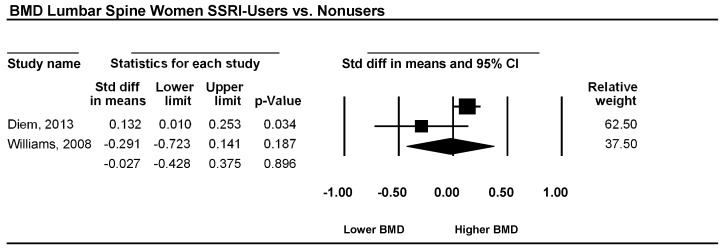
Estimates of all studies that compared bone mineral density (BMD) in the lumbar spine in women with and without treatment with antidepressive agents. The diamond at the bottom of the graph denotes the overall estimate of the association between the use of antidepressive agents and bone mineral density.

Two studies examined the lumbar spine in patients using an SSRI as the antidepressive agent versus a non-using control group. The pooled effect sizes for women, corresponding CI, *p*-values, and relative weights for each study and a forest plot summarizing the association between depression and BMD are shown in Figure 2. The effect sizes ranged from −0.29 to 0.13, with one study reporting either lower or similar BMD and one study showing higher BMD. However d, was −0.03, and its CI was between −0.43 and 0.38. This result implies that overall BMD is not significantly lower in women using SSRIs than in non-using women (*p* = 0.896). The heterogeneity was very low (I^2^ = 0).

### 3.2. Femoral Neck

**Figure 3:**
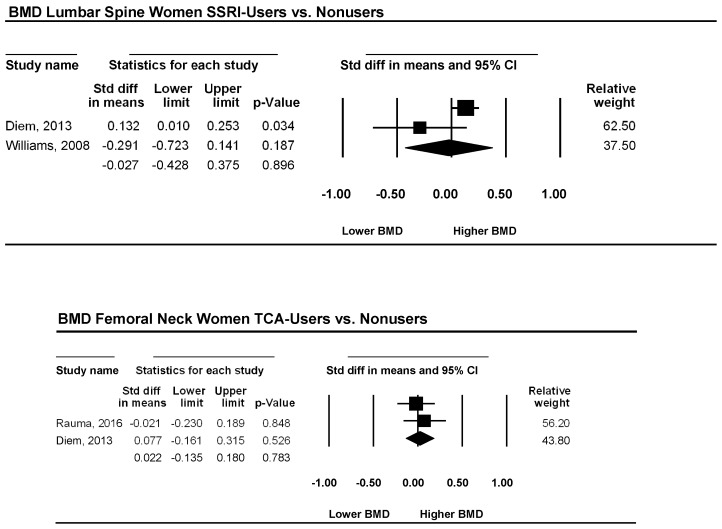
Estimates of all studies that compared bone density in the femoral neck in women with and without a treatment with antidepressive agents. The diamond at the bottom of the graph denotes the overall estimate of the association between the use of antidepressive agents and bone mineral density.

Three studies examined the femoral neck bone mineral density in patients using an SSRI as the antidepressive agent versus a non-using control group. The pooled effect sizes for women, corresponding CI, *p*-values, and relative weights for each study and a forest plot summarizing the association between depression and BMD are shown in Figure 3. The effect sizes ranged from −0.45 to 0.30. Two studies reported lower or similar BMD. One study reported higher BMD, d, was −0.06, and its CI was between −0.50 and 0.35. This finding suggests that overall BMD is not significantly lower in women using SSRIs than in non-using women (*p* = 0.777). The heterogeneity was very low (I^2^ = 5.6).

Two studies examined the femoral neck bone mineral density in patients using TCAs as an antidepressive agent versus a non-using control group. The pooled effect sizes for women, corresponding CI, *p*-values, and relative weights for each study and a forest plot summarizing the association between depression and BMD are shown in Figure 3. The effect sizes ranged from −0.02 to 0.08. One study reported lower or similar BMD. One study showed higher BMD, d, was 0.02, and its CI was between −0.14 and 0.18. This finding suggests that overall BMD is not significantly lower in women using TCAs than non-using women (*p* = 0.783). The heterogeneity was very low (I^2^ = 0).

### 3.3. Total Hip

**Figure 4:**
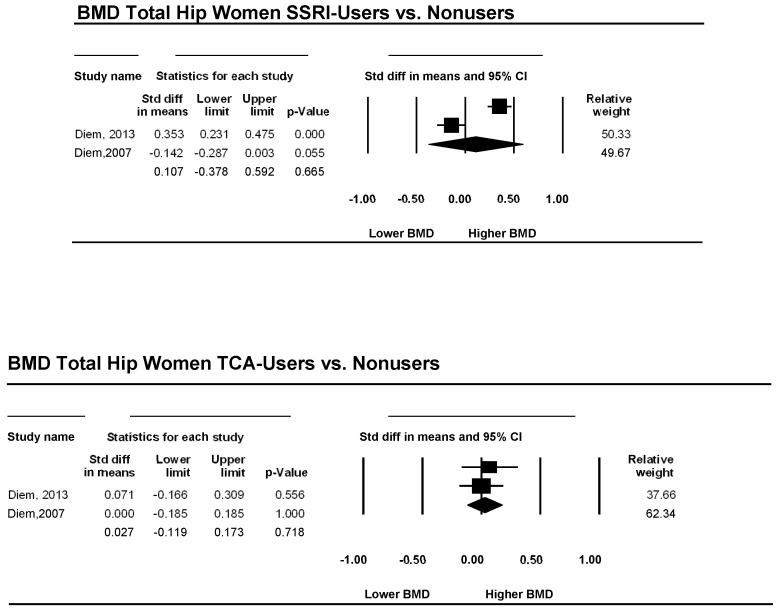
Estimates of all studies that compared bone mineral density in the hip in women with and without treatment with antidepressive agents. The diamond at the bottom of the graph denotes the overall estimate of the association between the use of antidepressive agents and bone mineral density.

Two studies examined the total hip bone mineral density in patients using an SSRI as an antidepressive agent versus a non-using control group. The pooled effect sizes for women, corresponding CI, *p*-values, and relative weights for each study and a forest plot summarizing the association between depression and BMD are shown in Figure 4. The effect sizes ranged from −0.14 to 0.35, with one study reporting either lower or similar BMD and one study showing higher BMD in patients using SSRIs. However, d, was 0.11, and its CI was between −0.38 and 0.59. This result implies that overall BMD is not significantly lower in women using SSRIs than in non-using women (*p* = 0.665). The heterogeneity was very low (I^2^ = 0).

Two studies examined the total hip bone mineral density in patients using TCAs as an antidepressive agent versus a non-using control group. The pooled effect sizes for women, corresponding CI, *p*-values, and relative weights for each study and a forest plot summarizing the association between depression and BMD are shown in Figure 4. The effect sizes ranged from 0.00 to 0.07, with one study reporting either lower or similar BMD and one study showing higher BMD in patients using TCAs. However, d, was 0.02, and its CI was between −0.12 and 0.17; this result implies that overall BMD is not significantly lower in individuals using TCAs than in non-using individuals (*p* = 0.966). The heterogeneity was very low (I^2^ = 0).

## 4. Discussion

This meta-analysis suggested that the association between antidepressant medication and bone mineral density in women has not been extensively researched. There were only four studies fulfilling our inclusion criteria. The global result of the meta-analysis was that the use of antidepressive agents is not associated with a lower or higher BMD. This result applied to both SSRIs and TCAs and to all measurement locations (lumbar spine, femoral neck, and total hip).

A possible biochemical explanation for the negative finding may be that the positive and negative effects on osteoblasts and osteoclasts are balanced within the patient cohorts. Antidepressive agents may decrease the activity of the HPA system [42] and decrease bone resorption by modulating this axis. This outcome may be particularly useful for the subgroup of patients with severe typical depression who suffer from considerable hypercortisolism. On the other hand, antidepressive agents may increase serotonin concentrations in the synaptic cleft and inhibit osteoblast proliferation in this way [34]. This result may be particularly relevant for patients who are not experiencing activation of the HPA system.

A close inspection of the studies showed that only one study limited itself to patients with a lifetime diagnosis of major depression assessed with a structured diagnostic interview [38]. All other studies used community-dwelling populations characterized by the absence or presence of antidepressive agent use. Only one study assessed the number of defined daily doses [40]. The individual doses were highly variable among the studies. According to the self-rating data, only a minority of the participants taking antidepressive agents were suffering from clinical depression at the time of the study [39,40], while current depressive status was not reported in the other two studies. When studying the association between depression and bone mineral density, it has to be kept in mind that cross-sectional studies do not allow for conclusions of causality. There were no randomized controlled studies on the effect of antidepressive agents on bone mineral density. There was one longitudinal observational study that reported increased bone loss with antidepressive agent use. We included the five year follow-up data in this study. Potential confounding factors for the association between depression and bone mineral density are the use of other drugs and an alteration of physical activity and nutrition due to depressive disorders.

Two population-based studies have reported an increased fracture risk in adults aged 50 years or older that used SSRI or Serotonin–norepinephrine reuptake inhibitors SNRI on a daily basis, after controlling for multiple risk factors [43,44]. When discussing these findings together with the results of our meta-analysis, it has to be taken into account that (i) antidepressants may alter attentional capacities and balance control [45] with the result of an increased risk of falls, and (ii) antidepressants may not only affect bone mineral density but also bone microarchitecture [46] reducing bone strength.

The limitations of this analysis are thus: because of the small number of studies identified, this meta-analysis is particularly vulnerable to publication bias. One major study may already have altered the overall result. The estimated effect measures and their corresponding confidence intervals of SSRIs and TCAs on bone mineral density ranged from small positive to medium negative effects. The heterogeneity for all meta-analyses were very low (I^2^ < 6), however this is dependent on the small amount of studies. The research strategy was limited to studies reporting bone mineral density and did not include research reporting fracture risk or data on bone microarchitecture. As stated above, bone mineral density cannot be simply equated with bone strength.

Given the high prevalence of antidepressive agent use, there is a need for prospective cohort studies with linkage to prescription registries as well as controlled trials in populations that are well characterized in terms of depressive disorder, bone mineral density and intensity of exposure to antidepressive agents. Such studies should include a broader spectrum of age groups. It may be useful to study the effects of antidepressant agents on bone microarchitecture using high-resolution peripheral quantitative computed tomography (HR-pQCT), which allows assessment of parameters of bone strength independent of bone mineral density [47].

## Figures and Tables

**Table 1:** Demographic and clinical characteristics of the included studies that compared bone mineral density, using dual-energy X-ray absorptiometry in women using antidepressive agents versus women without treatment.

Study Name	Year	Country	User/Nonuser Subjects (n)	Age (User/Nonuser)	Menopausal Status	Drugs	Bone Site
P.H. Rauma et al.	2016	Finland	210/1669	63.4/63.7	Post	SSRI, TCA, Others	Femur
S. J. Diem et al.	2013	US	382/1590	49.6/49.7	Pre/Post	SSRI, TCA	Lumbar, Femur, Hip
L.J. Williams et al.	2008	Australia	26/102	57.5/51	Pre/Post	SSRI	Lumbar, Femur
S. J. Diem et al.	2007	US	316/2406	78.4/78.6	Post	SSRI, TCA	Hip
